# Supplementary material for: A simulation modelling toolkit for organising outpatient dialysis services during the COVID-19 pandemic
Source: PLoS One. 2020 Aug 13;15(8):e0237628. doi: 10.1371/journal.pone.0237628 (PMC7425906; doi:10.1371/journal.pone.0237628)
Supplement: S1 Appendix — Overview of the dialysis network’s geographic setting. Tabular description of capacity at each facility and open sessions. (PDF) [file pone.0237628.s001.pdf]

A simulation modelling toolkit for organising outpatient dialysis services during the COVID-19 pandemic

Michael Allen[1], Amir Bhanji[2], Jonas Willemsen[2], Steven Dudfield[2], Stuart Logan[1], and Thomas Monks *[3]

[1]University of Exeter Medical School & NIHR South West Peninsula Applied Research Collaboration (ARC).
[2]Portsmouth Hospitals, NHS Trust
[3]University of Exeter Medical School

July 1, 2020

*Corresponding authors: m.allen@exeter.ac.uk and t.m.w.monks@exeter.ac.uk

Appendix S1: Study setting geography

This model focuses on the South of England including the towns and cities of Andover, Bognore Regis, Basingstoke, Portsmouth, Salisbury, Southampton, and Winchester. Our analysis includes includes 582 dialysis patients, from 262 postcode sectors, attending nine dialysis units. In this paper we do not include home dialysis patients 44 patients on the Isle of Wight.

Table 1 provides information on regional dialysis units.

Table 2 shows which sessions are currently used at each unit (the model maintains this pattern of open/closes sessions unless specified). Each session may be designated for with COVID-19 negative (or COVID-recovered) or COVID-positive patients. COVID-positive and COVID-non-positive patients never share a session. Units are designated as allowing sessions to be made COVID-positive. Where a unit may want to retain some capacity only for non-positive patients, the unit may be split into two (or more) sub-units which may each be designated as allowing switching to COVID-positive status.

Table S1: Dialysis units. Units may be split into sub-units if some, but not all, of the capacity, may be opened for use for COVID-positive patients. The *COVID order* shows the preferred order of opening up capacity for COVID-positive patients (only when one unit is at maximum capacity is the next unit opened).

Name	unit	subunit	Location	Chairs	inpatient	Allow COVID	COVID order
Basingstoke	BST	BST-1	RG21 6YH	12	-	-	-
Basingstoke	BST	BST-2	RG21 6YH	13	-	Y	2
Bognor Regis	BGN	BGN	PO22 9PP	13	-	-	-
Chandler's Ford	CHF	CHF	SO53 4DG	18	-	-	-
Havant	HAV	HAV	PO9 1TR	28	-	-	-
Milford-on-Sea	MIL	MIL	SO41 0PG	7	-	-	-
Queen Alexandra	HU	HU-1	PO6 3LY	12	Y	-	-
Queen Alexandra	HU	HU-2	PO6 3LY	12	-	Y	1
Salisbury	SAL	SAL	SP2 8BJ	11	-	-	-
Totton	TOT	TOT	SO40 3ZN	9	-	-	-

Table S2: Dialysis unit open sessions

Name	subunit	Mon 1	Mon 2	Mon 3	Tues 1	Tues 2	Tues 3
Basingstoke	BST-1	Y	Y	-	Y	Y	-
Basingstoke	BST-2	Y	Y	-	Y	Y	-
Bognor Regis	BGN	Y	Y	Y	Y	Y	-
Chandler's Ford	CHF	Y	Y	Y	Y	Y	-
Havant	HAV	Y	Y	Y	Y	Y	Y
Milford-on-Sea	MIL	Y	Y	-	Y	-	-
Queen Alexandra	HU-1	Y	Y	Y	Y	Y	Y
Queen Alexandra	HU-2	Y	Y	Y	Y	Y	Y
Salisbury	SAL	Y	Y	-	Y	Y	-
Totton	TOT	Y	Y	-	Y	Y	-
