# Supplementary material for: A simulation modelling toolkit for organising outpatient dialysis services during the COVID-19 pandemic
Source: PLoS One. 2020 Aug 13;15(8):e0237628. doi: 10.1371/journal.pone.0237628 (PMC7425906; doi:10.1371/journal.pone.0237628)
Supplement: S2 Appendix — Details of the vehicle routing algorithms implemented for the transport simulation modelling. (PDF) [file pone.0237628.s002.pdf]

A simulation modelling toolkit for organising outpatient dialysis services during the COVID-19 pandemic.

Michael Allen[1], Amir Bhanji[2], Jonas Willemsen[2], Steven Dudfield[2], Stuart Logan[1], and Thomas Monks *[3]

[1]University of Exeter Medical School & NIHR South West Peninsula Applied Research Collaboration (ARC).
[2]Portsmouth Hospitals, NHS Trust
[3]University of Exeter Medical School

July 1, 2020

*Corresponding authors: m.allen@exeter.ac.uk and t.m.w.monks@exeter.ac.uk

Appendix S2: Vehicle routing methods

Model formulation

Our primary solution method is via meta-heuristic. However, for clarity we formulate the CVRP as a Mixed Integer Programme (MIP). Note that it is possible to use exact methods to solve the MIP in small to medium instances. We tested this approach using the industrial solver Gurobi - a modern powerful solver that exploits parallelism. For instances as small as 15 patients we found that the optimality gap between was a high as %40 after 2.5 hours of runtime (although we note that a time limit and/or performance tweaks could be made to Gurobi). We also found that the average performance of Iterated Local Search matched or exceeded Gurobi on larger instances (40+ patients) when the runtime was seconds as opposed to hours.

If:

n is the number of patients

N is the set of patients $\{1, 2, ..., n\}$

V is the set of vertices (nodes) to visit. $V = \{0\} \cup N$

A is the set of arcs (links) $A = \left\{(i, j) \in V^2 : i \neq j\right\}$

c_{ij} is cost of travel over arc $(i, j) \in A$

Q is the vehicle capacity (for patient transport this is an integer)

q_i is the load represented by a patient (for patients this is always an integer = 1)

$$
\begin{aligned}
\min & \sum_{i=1}^{n}\sum_{j=1}^{n} c_{ij}x_{ij} & \\
\text{s.t.} & \sum_{i=1}^{n} x_{ij} = 1, & j \in N, j \neq i \\
& \sum_{j=1}^{n} x_{ij} = 1, & i \in N, i \neq j \\
& \text{if } x_{ij} = 1 \ \Rightarrow \ u_i + q_j = u_j & i, j \in A : j \neq 0, i \neq 0 \\
& q_i \leq u_i \leq Q & i \in N \\
& x_{ij} \in \{0, 1\} & i, j \in A
\end{aligned}
$$

Clarke-Wright Savings

Assume there are two patients A and B, and a transport vehicle is with 2 seats is based at a depot D.

- The the time to travel from D to A is 30 minutes,
- The time to travel from D to B is 40 minutes
- The travel time from A to B is 10 minutes.

If single trips are used the the total time needed to transport all patients to hospital is $2(30) + 2(40) = 140$ minutes.

If the capacity of an ambulances is increased to two seats then **the saving in time relative to single trips** is $30 + 40 - 10 = 60$ minutes. I.e $D \rightarrow A \rightarrow B \rightarrow D$ is one trip from D to A (30 minutes) + one trips from B to D (40 minutes) minus the time to travel from A to B (10 minutes).

The algorithm calculates these savings for all combinations of patient locations. It constructs routes by selecting the locations with the highest saving first. In the sequential version of the algorithm additional adjacent links are added again prioritised by savings.

Iterated Local Search

Iterated Local Search (ILS) is a meta-heuristic designed to overcome the problem of hill-climbing algorithms becoming stuck in local optima (good solutions, that are not the global optimum or best). ILS runs hill-climbing algorithms multiple times and stochastically climbs (or descends) the hill of local-optima. Algorithm 1 describes our implementation of the standard ILS procedure. Our initial solution was fed through from the Clarke-Wright Savings procedure. For each problem instance we iterated 20 times over a first improvement decent local search procedure that employed 2-Opt swaps of patient allocations to routes. To balance exploitation and exploration of the space of local optima, we use an Epsilon-Greedy (ϵ = 0.2) implementation of the *homebase* function (see algorithm 2). Our perturbation function employed a 4-Opt (the Double-Bridge) swap.

Algorithm 1: Iterated Local Search

Given P patient locations and n iterations to run.
$S \leftarrow SequentialClarkeWrightSavings(P)$
$H \leftarrow S$
$Best \leftarrow S$
for $i <= n$ **do**
  $S \leftarrow LocalSearch(Copy(S))$
  if $Quality(S) > Quality(Best)$ **then**
    $Best \leftarrow S$
  $H \leftarrow NewHomeBase(H, S)$
  $S \leftarrow Perturbation(H)$
return $Best$

Algorithm 2: Epsilon-Greedy $NewHomeBase$

Given ϵ, H and S
$u \leftarrow Uniform(0, 1)$
if $u > \epsilon$ **then**
  if $Quality(S) > Quality(H)$ **then**
    return S
  else
    return H
else
  return S
